# Emergence of High Prevalence of Extended-Spectrum Beta-Lactamase and Carbapenemase-Producing *Enterobacteriaceae* Species among Patients in Northwestern Ethiopia Region

**DOI:** 10.1155/2022/5727638

**Published:** 2022-02-04

**Authors:** Selamyhun Tadesse, Wondemagegn Mulu, Chalachew Genet, Mulugeta Kibret, Melaku Ashagrie Belete

**Affiliations:** ^1^Department of Medical Laboratory Science, College of Health Sciences, Woldia University, Ethiopia; ^2^Departments of Medical Laboratory Science, College of Medicine and Health Sciences, Bahir Dar University, Ethiopia; ^3^Department of Biology, Science College, Bahir Dar University, Ethiopia; ^4^Department of Medical Laboratory Science, College of Medicine and Health Sciences, Wollo University, Dessie, Ethiopia

## Abstract

**Background:**

World Health Organization identified some *Enterobacteriaceae* as superbugs because of their high production and spread of extended-spectrum beta-lactamases (ESBL) and carbapenemases. Moreover, their resistance against commonly prescribed antibiotics left few choices of drugs to treat infection. This study is aimed at determining the magnitude of ESBL and carbapenemase-producing *Enterobacteriaceae* pathogens and their antimicrobial resistance pattern.

**Materials and Methods:**

A hospital-based cross-sectional study was carried out from February to April 2019 in the Northwestern Ethiopia region. A total of 384 patients presumptive for bacterial infections were conveniently enrolled in the study. Specimens were collected and processed following standard bacteriological procedures. Drug susceptibility tests were performed using disk diffusion technique. ESBL and carbapenemase enzymes were tested by double disk diffusion and modified carbapenem inhibition methods, respectively. The data obtained were analyzed using SPSS version 22 software, and descriptive statistics were summarized in tables and graphs.

**Results:**

Out of 384 clinical specimens processed 100 (26%) were culture positive for *Enterobacteriaceae*. The proportion of *Enterobacteriaceae* infection was relatively higher among in-patients 86 (32.6%) than out-patients 14 (11.7%). Overall, *Escherichia coli* 35 (9.1%) was the leading isolate followed by *Klebsiella pneumoniae* 31 (8.1%). *Klebsiella pneumoniae* 15 (15.6%) was the most frequent isolate from bloodstream infection and is the leading isolate from intensive care unit patients 15 (38.3%). Overall, 44 (44%) of *Enterobacteriaceae* were extended-spectrum beta-lactamase producers. Among them, *Citrobacter* spp. was the leading one 4 (80%) followed by *Enterobacter cloacae* 6 (60%) and *K*. *pneumoniae* 18 (58.1%). Furthermore, 6 (6%) of *Enterobacteriaceae* were carbapenemase-producers, in which 5 (50%) of *E*. *cloacae* and 3 (9.7%) of *K*. *pneumoniae* had highest percentage*. Conclusions*. ESBL and carbapenemase-producing isolates of *Enterobacteriaceae* are alarmingly spreading in the study area. Thus, improving the infection prevention strategy and further screening at the national level is recommended to develop the optimal use of antibiotics.

## 1. Background

Bacterial resistance to antibiotics has become a major global health issue worldwide. It is associated with widespread use, misuse, and nonmedical use of antibiotics [1]. The rising level of antibiotic resistance is a particular concern in *Enterobacteriaceae*. Carbapenem and third-generation cephalosporin-resistant *Enterobacteriaceae* are on World Health Organization's (WHO) list of top-priority pathogens, which new antibiotics are needed for their extensive resistance [2].

Majority of the *Enterobacteriaceae* are normal inhabitants of the human gastrointestinal tract, and infections they cause occur due to displacement from their natural habitation [3]. They cause different infections including bloodstream infection (BSI), urinary tract infection (UTI), and lower respiratory tract infections (LRTIs), and wound infection. Such infections are primarily treated with beta- (*β*-) lactam class of antibiotics such as penicillins, cephalosporins, monobactams, and carbapenems [4, 5].

The development of antimicrobial resistance (AMR) among *Enterobacteriaceae* is mediated by both enzymatic and nonenzymatic mechanisms. The genes involved in the resistance may be intrinsic or acquired [6]. Beta-lactamases are enzymes produced by *Enterobacteriaceae* that can hydrolyse the *β*-lactam ring of antibiotics. Extended-spectrum *β*-lactamases (ESBL) are enzymes that can hydrolyse and mediate resistance to penicillins, first-, second-, and third-generation cephalosporins, and monbactams. Carbapenemases have a wide hydrolysing activity that can degrade all *β*-lactams including carbapenems and *β*-lactam inhibitors like clavulanic acid, sulbactam, and tazobactam [7].

The genes that code for ESBL and carbapenemase are found on mobile genetic elements and are important in the spread of drug resistance. Moreover, they allow *Enterobacteriaceae* to acquire resistance to other classes of antibiotics such as aminoglycosides, sulfamethoxazole-trimethoprim, and quinolones [8, 9]. Thus, the emergence of ESBL and carbapenemase-producing (CP) isolates has important clinical and therapeutic implications [7]. Carbapenems are the last-resort antibiotics for treating infections due to *Enterobacteriaceae*. Resistance to carbapenems in these species is related to overexpression of ESBL together with efflux pumps, impermeability, or expression of carbapenem-hydrolysing carbapenemase [10].

Extensive use and misuse of antibiotics in clinical, environmental, and agricultural areas, empirical treatment, and taking a drug without prescription are the major attributable factors to the emergence of beta-lactamase-mediated resistance [11, 12]. Furthermore, prolonged hospital stay, underline medical conditions, and invasive procedures significantly contribute to the rise of resistance [7].

ESBL and carbapenemase-mediated resistance increase the length of hospital stay, raise health care costs, and are significant predictors of mortality [13–15]. According to communicable disease control, 2013 report, annually ESBL-producing and carbapenem resistance (CR) *Enterobacteriaceae* caused 35,000 morbidities and 2,000 mortalities in the United States [1].

Nowadays, ESBL and CP *Enterobacteriaceae* are increasingly being identified worldwide and are becoming hot clinical issues. Thus, the evolution of these organisms from multiple resistances to pan-drug resistance is the future threat. Therefore, data on *β*-lactamase-producing *Enterobacteriaceae* is indispensable, and this is the only way to maintain the efficacy of the last resort antibiotics [7].

However, there is a scarcity of data in Ethiopia, especially in the study area. Therefore, a study on ESBL and carbapenemase production in *Enterobacteriaceae* among patients is essential to generate baseline data, for guiding local empirical therapy, planning local infection control programs, and developing antimicrobial prescription protocols for different infections. Thus, this study is aimed at determining the magnitude of ESBL and CP *Enterobacteriaceae* isolated from patients presumptive for different bacterial infections.

## 2. Materials and Methods

### 2.1. Study Design, Period, and Setting

A hospital-based cross-sectional study was conducted from February to April 2019 at Felege Hiwot Comprehensive Specialized Hospital (FHCSH), Northwestern Ethiopia. Felege Hiwot Comprehensive Specialized Hospital is a tertiary hospital that provides referral health care services for more than 7 million people. It has 430 beds in medical, surgical, orthopedics, and pediatrics wards and adult and neonatal intensive care units with 531 healthcare professionals. The daily outpatient clients are more than 600.

### 2.2. Sample Size Determination and Sampling Technique

The sample size was determined using Epi info version 3.5.1 (public domain software, http://www.cdc.gov) by considering a 95% confidence level and marginal error (5%) and a proportion of 0.5 ESBL. Thus, the total sample size was obtained to be 384. All patients who were clinically presumptive for different bacterial infections were included conveniently until the required sample size was achieved, and study participants who did not provide complete data and appropriate specimen (saliva and/or contaminated sputum) and insufficient volume of all specimens were excluded.

### 2.3. Data Collection

Data on demographic variables were gathered through face-to-face interviews using a structured questionnaire, complemented with a review of patients' medical records. The *Enterobacteriaceae* isolates, results of antimicrobial susceptibility testing of the isolates, and their profile of ESBL and CP were recorded by using a separate data collection worksheet.

### 2.4. Specimen Collection and Processing

All appropriate specimens were collected from study participants using leak-proof containers by strictly following standard microbiological procedures [16].

Urine sample: from the collected 10 ml of a freshly voided midstream urine sample, 0.001 ml was inoculated into Cysteine Lactose Electrolyte Deficient Medium (HiMedia, India) using a calibrated wire loop. Inoculated plates were incubated at 37°C for 24 hr. Colony counts yielding bacterial growth of ≥10^5^ CFU/ml of urine (significant bacteriuria) from CLED medium were then subcultured into MacConkey agar (MAC) (HiMedia, India) and blood agar (BA) plates (HiMedia, India) and then incubated at 37°C for 24 hr under aerobic atmosphere [16].

Blood sample: 10 ml of venous whole blood from adults, 5 ml from children, and 2 ml from neonates have been collected aseptically. Immediately after collection, the blood was added to tryptic soy broth and incubated at 37°C. If visible growth was observed, subculturing was made onto MAC agar and BA plate. When no growths were observed, the tryptic soy broth was further incubated for 7 days before being reported as negative [17].

Sputum sample: after briefly instructing the patients to rinse their mouths with water, a sterile wide-mouth container was used to collect 2 ml purulent sputum. Sputum specimens with much watery saliva were excluded from being processed using microbiological procedures. The sputum was immediately smeared and examined for appropriateness for culturing. Specimens that had more than 25 polymorphonuclear leukocytes and less than 10 epithelial cells were inoculated to MAC agar and BA plate and incubated for 24 hr at 37°C [16].

Wound sample: purulent exudates, pus, and discharges were collected aseptically from the depth of the wound using a syringe or sterile cotton swab. The cotton swab was immersed in a tube of brain heart infusion transport medium. The brain-heart infusion culture was incubated for 24 hr and then subcultured onto MAC agar and BA plates and reincubated for 24 hr at 37°C.

### 2.5. Identification of Bacterial Isolates

All *Enterobacteriaceae* isolates were identified using standard microbiological laboratory methods [17]. To identify *Enterobacteriaceae* species, colony characteristics and a panel of biochemical tests, including indole production, H_2_S production in triple sugar iron agar, citrate utilization, urease test, motility test, oxidase, and carbohydrate utilization tests, were used.

### 2.6. Antimicrobial Susceptibility Testing

Antimicrobial susceptibility to all identified bacterial isolates was performed using Kirby-Bauer disk diffusion method recommended by the Clinical and Laboratory Standards Institute (CLSI) [18]. Pure culture colonies of 24 hrs growth were suspended in a tube with 4 ml of physiological saline to get bacterial inoculums equivalent to 0.5 McFarland turbidity standard. A sterile cotton swab was used to evenly inoculate the colony suspension onto Muller-Hinton agar (HiMedia, India), and then, the antibiotic discs were placed on MHA plates.

All isolates were tested against the following antibiotic disks: penicillin (amoxicillin (10 *μ*g)), *β*-lactam/*β*-lactamase inhibitor combination (amoxicillin-clavulanic acid (20/10 *μ*g)), foliate pathway inhibitor (sulphamethoxazole-trimethoprim (1.25/23.75 *μ*g)), third-generation cephalosporins (cefoxitin (30 *μ*g), ceftazidime (30 *μ*g)), aminoglycosides (gentamicin (10 *μ*g)), fluoroquinolones (ciprofloxacin (5 *μ*g)), carbapenem (meropenem (10 *μ*g), ertapenem (10 *μ*g)), nitrofurantoin (30 *μ*g), and chloramphenicol (30 *μ*g) (Abtek Biologicals, UK). The plates were then incubated at 37°C for 24 hrs. The diameters of the zones of inhibition around the disks were measured using a digital caliper. The interpretation of the antimicrobial susceptibility test results as sensitive, intermediate, or resistant was based on the standardized chart supplied by CLSI guidelines [18]. Multidrug resistance enterobacteriaceae was checked if the isolate was nonsusceptible to at least 1 agent in ≥3 antimicrobial categories.

### 2.7. Screening for ESBL Production

Initial screening for ESBL was done by measuring the diameters of zones of inhibition produced by either ceftazidime (30 *μ*g) or cefotaxime (30 *μ*g) on the antimicrobial susceptibility test on MHA according to the criteria recommended by CLSI. The breakpoints indicative of suspicion for ESBL production was ≤22 mm for ceftazidime and ≤27 mm for cefotaxime [18].

### 2.8. Confirmation of ESBL Production

After initial screening, ESBL production was confirmed by combined disk method according to CLSI guidelines [18]. The organism to be tested was uniformly inoculated onto Mueller-Hinton Agar plate. Ceftazidime (30 *μ*g) and cefotaxime (30 *μ*g) disks alone and in combinations with clavulanic acid (30 *μ*g/10 *μ*g) were used at the same time for phenotypic confirmation of the presence of ESBLs. These four discs were placed at a distance of 25 mm apart on a Muller Hinton agar plate inoculated with bacterial suspension of 0.5 McFarland turbidity standards and incubated overnight (24 hrs) at 37°C. An increase in diameter of zone of inhibition ≥5 mm for either the ceftazidime-clavulanate and/or cefotaxime-clavulanate disk combination compared with the zone diameter of the respective cephalosporin disks alone was considered positive, and the isolate was interpreted as ESBL producer [18].

### 2.9. Detection of Carbapenemase Production

Bacterial isolates with fully or intermediate resistance to at least one of the carbapenems (ertapenem (10 *μ*g), and meropenem (10 *μ*g)) in the above disk diffusion test were further tested for the production of carbapenemase using modified carbapenem inhibitory method (mCIM) which is recommended by CLSI [18]. Accordingly, after the bacterial isolates were emulsified in 2 ml tryptic soya broth, meropenem disk (10 *μ*g) was added and then incubated at 37°C in ambient air for 4 hrs. McFarland standard equivalent suspension of carbapenem sensitive indicator organism (*E*. *coli* ATCC 25922) evenly swabbed to Mueller-Hinton Agar, and then, the meropenem in the tryptic soy broth was dispensed. After incubation for 24 h at 37°C, the zone of inhibition for meropenem was measured. If the bacterial isolate has a zone of inhibition of 6-15 mm or pinpoint colonies within a zone of inhibition of 16-18 mm and no inhibition of the carbapenem-susceptible *E. coli* ATCC 25922, it was considered as carbapenamase producer [18].

### 2.10. Quality Assurance

The data were checked for completeness and adequate recording on the worksheet during and after data collection. All laboratory assays were carried out by strictly maintaining quality control procedures. The sterility of the media was checked by incubating 5% of the batch at 35-37°C overnight. The expiry date of the media, reagents, and antibiotic disks were checked before use. Reference strains of *S. aureus* (ATCC 25923), *E. coli* (ATCC 25922), *E. faecalis* (ATCC 29212), and *P. aeruginosa* (ATCC 27853) were used as quality control throughout the study to check the abilities of the prepared media supporting bacterial growth for culture and susceptibility testing. For ESBL detection, *E. coli* ATCC 25922 and *K*. *pneumoniae* ATCC 700603 were used as positive and negative controls, respectively. *Klebsiella pneumoniae* ATCC BAA1705 and *K*. *pneumoniae* ATCC BAA1706 were used as positive and negative quality control strains for carbapenemase detection, respectively. The results were interpreted in accordance with the CLSI guidelines [18].

### 2.11. Data Analysis

The data generated were entered into the Epi-data version 3.1 every day, then were imported and analyzed by Statistical Package for Social Science (SPSS) version 22.0 (IBM USA). Descriptive statistics were calculated and summarized in graphs and tables to show the frequency of demographic characteristics, the magnitude of *Enterobacteriaceae* infections, and drug resistance profiles.

### 2.12. Ethics Approval

The study protocol was approved by the Institutional Review Board of College of Medicine and Health Sciences, Bahir Dar University, and ethical clearance was obtained with approval number CMHS/IRB 03-008. All methods involving humans were carried out following the relevant guidelines and regulations. A permission letter was obtained from Amhara Public Health Institute and FHCSH before data collection. Moreover, before commencing the study, written informed consent was obtained from each participant. Written assent was also obtained from parents and surrogates to obtain information from children and those who cannot give data. Subject confidentiality was kept by giving only codes for questionnaires. Participants who tested positive for the pathogen were reported to physicians for treatment and any other necessary care. Moreover, this study was conducted in accordance with the Declaration of Helsinki.

## 3. Results

### 3.1. Demographic Characteristics

A total of 384 patients symptomatic for bacterial infections from different sites took part in the study. Among them, 210 (54.7%) were males. The age of the study participants ranged from 0.02 to 80 years, with a median age of 32. Moreover, majority (58.4%) of the participants were rural dwellers. Two hundred and sixty-four (68.7%) were in-patients. Forty-two (10.9%) and 37 (9.6%) participants were from adults and neonatal ICUs, respectively (Table 1).

### 3.2. *Enterobacteriaceae* Infections

**Figure 1:**
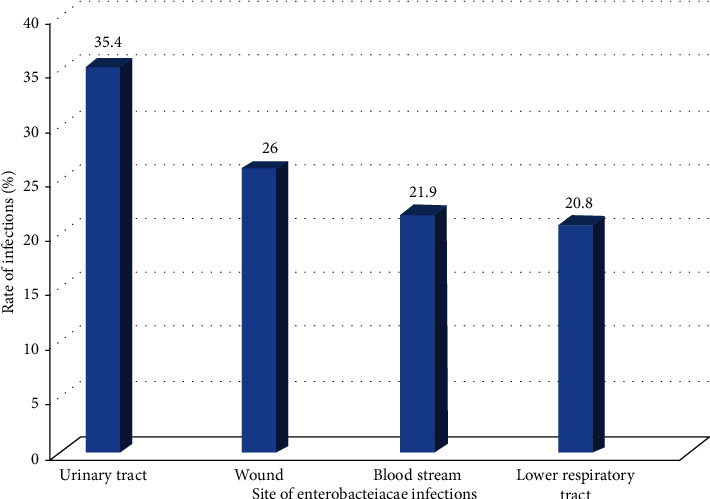
Proportion of culture confirmed *Enterobacteriaceae* infections among patients in Northwestern Ethiopia region, 2019.

Of the 384 patients, 100 (26.0%) were culture positive for *Enterobacteriaceae.* The proportion of *Enterobacteriaceae* infection was 86 (32.6%) for in-patients and 14 (11.6%) for out-patients. The proportion of infection was significantly higher among urban than rural dwellers (41.3% vs. 15.2%) (29.5% vs. 21.2%) (Table 1). The proportions of culture confirmed UTI, wound infection, BSI, and LRTI were 35.4%, 26%, 21.9%, and 20.8%, respectively (Figure 1).

### 3.3. Proportion of *Enterobacteriaceae* Isolates

Overall, *Escherichia coli* was the most common 35 (9.1%) isolate followed by *K. pneumoniae* 31 (8.1%) and *Enterobacter cloacae* 10 (2.6%). Moreover, *E*. *coli* was the leading isolate from UTI 21 (21.9%) followed by *K*. *pneumoniae* 7 (7.3%). On the other hand, *K*. *pneumoniae* was the most frequent isolate from BSI 15 (15.6%) followed by *E*. *coli* and *K*. *oxytoca* each accounted 2 (2.1%) (Table 2).

### 3.4. ESBL and Carbapenemase Production Profiles of the Isolates

**Figure 2:**
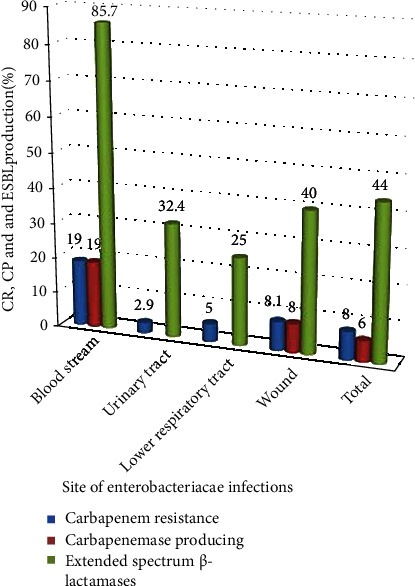
Carbapenem resistance, carbapenemase, and ESBL production profiles of *Enterobacteriaceae* isolates from different sites of infection in Northwestern Ethiopia region, 2019.

As shown in Table 3, overall, 44 (44%) of the *Enterobacteriaceae* isolates were ESBL producers. Among them, 4 (80%) of Citrobacter spp. and 18 (58.1%) of *K. pneumoniae* showed highest ESBL production. Among the *Enterobacteriaceae* isolates, 8 (8%) were CR, and 6 (6%) were CP. *Enterobacter cloacae* (50%), *K. pneumoniae* (9.7%), and *E. coli* (2.9%) were the identified carbapenemase producers. All CR *K. pneumoniae* isolates were found to be CP producers (Table 3). Carbapenem resistance was higher in blood 4 (19%) than in other specimens (19% vs. 2.9-8.1%). At the same time, the frequency of ESBL-producing isolates was higher in blood than in other samples (85.7% vs. 25-40%) (Figure 2).

### 3.5. Antibiotic Resistance Profiles of *Enterobacteriaceae* Isolates

The majority of *Enterobacteriaceae* isolates exhibited the highest resistance rate to amoxicillin 87 (87%), amoxicillin-clavulanic acid 74 (74%), and sulfamethoxazole–trimethoprim 62 (62%). Among the isolates, *E. coli* revealed a high level of resistance to amoxicillin 28 (80%) and amoxicillin-clavulanic acid 22 (62.9%). *K. pneumoniae* isolates exhibited 30 (96.8%) and 26 (83.9%) rates of resistance to amoxicillin and amoxicillin-clavulanic acid, respectively. Regarding the level of resistance against third-generation cephalosporins, most of the isolates were resistant to cefotaxime (58%) and ceftazidime (57%). The highest rate of resistance to cefotaxime and ceftazidime was found in *E*. *cloacae* (90% and 80%) and *Citrobacter* spp. each accounted for 80% (Table 4).

## 4. Discussion

The emergence of ESBL-producing and CR *Enterobacteriaceae* isolates has important clinical and therapeutic implications that limit the treatment options for infected patients [3, 19, 20].

The present study finding confirmed that *Enterobacteriaceae* are the major pathogens which cause different body sites of infections in both hospitalized and ambulatory patients. The proportion of *Enterobacteriaceae* infection in the study area was relatively higher in urban than rural residents. This might be related with variations in patients' exposure to antibiotics which is a major factor for selection of drug resistance. Moreover, the proportion of infection was relatively higher among in-patients than out-patients, which could be attributed to the fact that admitted patients are more likely to be exposed to these pathogens from other patients, health care workers, and hospital environments through cross infection.

In the present study, *Escherichia coli* was the predominant *Enterobacteriaceae* isolate, followed by *K*. *pneumonia* and *E. cloacae*. This is concurrent with findings from other studies in Ethiopia [19, 20], Burkina Faso [21], and Uganda [22]. While *E*. *coli* followed by *K*. *pneumoniae* and *Proteus* spp. were the most common isolates from UTI, *K. pneumoniae* followed by *E*. *coli* were the most common isolates from BSI. This is also consistent with other reports from Ethiopia [20, 23, 24]. This could be because *E*. *coli* and *K*. *pneumoniae* are abundant normal flora of gastro-intestine, which in turn via contamination ascends through different routes to cause infections. Furthermore, their fimbriae structure can mediate colonization and invasion of various sites [3].

The magnitude of ESBL-producing *Enterobacteriaceae* isolates (44%) in this study is in line with studies from Iran 40.8% [25] and India 48.27% [26]. However, variable results were documented from different parts of Ethiopia (28.2% and 78.6%) [23, 27] and Burkina Faso (58%) [21]. *Citrobacter* spp. were the most prevalent ESBL producer in this study (80%), which was higher than reports from Ethiopia (54.5%) [19] and Burkina Faso (58%) [21]. Moreover, the magnitude of ESBL among *K*. *pneumoniae* isolates in this study (58.1%) agrees with a study from Burkina Faso (66%) [21]. A variable prevalence of ESBL production among *K*. *pneumoniae* was reported from other studies in Ethiopia (70.4-84.2%) [23, 24, 27] and elsewhere (12.3-41.4%) [22, 25, 26]. The magnitude of ESBL-producing *E*. *coli* isolates in this study (37.1%) is in line with a study done in Benin (36.25%) [28]. However, higher results were reported from previous studies in Ethiopia (45% and 60%) [19, 24], Burkina Faso (60%) [21], India (77.3%) [26], and Iran (52.9%) [25]. Conversely, lower results were documented in Uganda (28.1%) [22]. The higher prevalence of ESBL from different studies might be due to higher colonization of *Enterobacteriaceae* in hospitals, which in turn increases the spread of ESBL genes in health care associated strains [7]. Although there are variations in the magnitude of ESBL, all findings collectively showed a rise in ESBL-producing isolates in developing countries, which might be attributed to widespread use of cephalosporins, poor control of antibiotic utilization, and empirical therapy.

The magnitude of CR among *Enterobacteriaceae* isolates in the present study was 8%. This is in line with a report from Addis Ababa, Ethiopia (12.1%) [23]. However, 0.96% to 35% CR was reported in other countries of East Africa [29]. This indicates that CR is alarmingly increasing time to time, which is a major concern for developing countries like Ethiopia as these drugs are the last sort for the treatment of superbugs and are still not routinely prescribed.

The rate of CR in *E*. *cloacae* (30%), *K*. *pneumoniae* (9.7%), and *E*. *coli* (5.7%) in the present study is higher than a previous report in Ethiopia, which were 6.3%, 6.9%, and 1.8%, respectively [20]. This indicates that CR is increasing in those species over time. However, this finding is inconsistent with a report from other parts of Ethiopia, where 16.7%, 30%, and 16.1%, rates of CR were documented for *E*. *cloacae*, *K*. *pneumoniae*, and *E*. *coli*, respectively [19]. The reason might be the later study was done exclusively among patients presumptive for nosocomial infection, which in turn increases the rate of CR.

The resistance of *Enterobacteriaceae* to carbapenem is mainly mediated by carbapenemase production [30]. In this study, 6% of *Enterobacteriaceae* isolates were CP. A relatively lower result was documented from a previous study in Ethiopia (2.7%) [20]. The increase in carbapenemase production could be due to a difference in study period. Besides, in the present study, 75% of CR isolates were CP. This supports the fact that CP-related mechanisms of resistance are highly prevalent in *Enterobacteriaceae* in the study area. This is a serious condition because CP-related resistance mechanisms are mostly plasmid-mediated, more easily spread, and the resistance determining genes can spread within enteric bacterial species and across species [7].

In this study, a significant proportion of CP-producing *E*. *cloacae* (20%), *K*. *pneumoniae* (9.7%), and *E*. *coli* (2.9%) was found. This is consistent with a systematic review report from East Africa, where a high rate of CP-producing *K*. *pneumoniae* and *E*. *coli* was documented in eight different studies [29].

Bacterial pathogen resistance to the commonly used antibiotics left clinicians with very limited choices of drugs for the treatment of various diseases [12]. In the present study, *Enterobacteriaceae* isolates revealed high levels of resistance to amoxicillin (87%), followed by amoxicillin-clavulanic acid (74%) and sulfamethoxazole-trimethoprim (62%). This might be due to the wide availability and the blind prescription of these antibiotics in the areas.

In our study, *E*. *coli* showed a considerable level of resistance to amoxicillin-clavulanic acid (62.9%), ceftazidime (40%), and cefotaxime (40%). Conversely, a higher level of resistance was reported in studies from Jimma, Ethiopia, for amoxicillin-clavulanic acid (90.3%) [31], Benin for amoxicillin-clavulanic acid (85.7%) and cefotaxime (56.5%) [28], and India for cefotaxime (76.3%) and ceftazidime (64.5%) [26]. The difference in resistance rate may be due to the loose prescription protocol of the antibiotics in those study areas. However, a comparable rate of resistance to ceftazidime (33%) and cefotaxime (31%) was documented in Rwanda [32].

*Klebsiella pneumoniae* also showed a higher level of resistance to amoxicillin-clavulanic acid (83.9%), cefotaxime (67.7%), and ceftazidime (70.1%) in the present study. This is variable with other studies in Ethiopia where 60.7% and 89.5% rate of resistance for amoxicillin-clavulanic acid, 28.6% and 100% for cefotaxime [20, 23], and 57.1% for ceftazidime.

Moreover, *Enterobacteriaceae* are known to acquire resistant plasmids from intra- and interspecies, which is important for obtaining resistance genes for multiple antimicrobial agents [7].

Due to the limitations of the laboratory facility, the different ESBL and carbapenemase determining genes were not detected.

## 5. Conclusions

Carbapenem resistance linked with a high rate of MDR, carbapenemase, and extended-spectrum beta-lactamase-producing isolates is alarmingly increasing among isolates of *Enterobacteriaceae* in the study area. Resistance to third generation cephalosporins is also a critical problem. Therefore, improving the infection prevention strategy and further national surveillance on the profile of carbapenem resistance, carbapenemase, and ESBL production and their determining genes among *Enterobacteriaceae* clinical isolates is required to adjust the routine use of antimicrobials.

## Figures and Tables

**Table 1:** Culture confirmed *Enterobacteriaceae* infections from different clinical samples among patients (*n* = 384) in Northwestern Ethiopia region, 2019.

Variables	Culture positive *N* (%)	Culture negative *N* (%)	Total *N* (%)
Age (years)			
0.002-0.02	4 (22.2)	14 (77.7)	18 (4.7)
0.021-5	11 (57.9)	8 (42.1)	19 (4.9)
6-18	5 (11.1)	40 (88.9)	45 (11.7)
19-28	14 (22.2)	49 (77.8)	63 (16.4)
29-38	25 (30.9)	56 (69.1)	81 (21.1)
39-48	14 (25.5)	41 (74.5)	55 (14.3)
49-58	13 (28.9)	32 (71.1)	45 (11.7)
>58	14 (36.8)	24 (63.2)	38 (9.9)
Gender			
Male	53 (25.2)	157 (74.8)	210 (54.7)
Female	47 (27)	127 (73)	174 (45.3)
Residence			
Urban	66 (29.5)	158 (70.5)	224 (41.6)
Rural	34 (21.2)	126 (78.7)	160 (58.4)
Wards of patients			
ICU	14 (33.3)	28 (66.7)	42 (10.9)
NICU	9 (24.3)	28 (75.7)	37 (9.6)
Medical	36 (41.9)	50 (58.1)	86 (22.4)
Surgery	11 (28.9)	27 (71.1)	38 (9.9)
Orthopedics	10 (32.3)	22 (71)	31 (8.1)
Pediatrics	6 (20.0)	24 (80.0)	30 (7.8)
OPD	14 (11.7)	106 (88.3)	120 (31.3)
Hospital patient setting			
In-patients	86 (32.6)	178 (67.4)	264 (68.7)
Out-patients	14 (11.7)	106 (88.3)	120 (31.3)
Total, *N* (%)	**100 (26)**	**284 (74)**	**384 (100)**

Key: ICU: intensive care unit; NICU: neonatal intensive care unit; OPD: outpatient department.

**Table 2:** Frequency of *Enterobacteriaceae* species in clinical samples collected from different sites of infection among patients in Northwestern Ethiopia region, 2019.

Variables	Enterobacteriaceae species							
	*E. coliN* (%)	*K. pneumoniaeN* (%)	*K. ozaenaeN* (%)	*K. oxytocaN* (%)	*E. cloacaeN* (%)	*Citrobacter* spp. *N* (%)	*Proteus* spp. *N* (%)	*Providencia* spp. *N* (%)
Type of specimen								
Urine (*n* = 96)	21 (21.9)	7 (7.3)	1 (1)	1 (1)	0	1 (1)	2 (2.1)	1 (1)
Blood (*n* = 96)	2 (2.1)	15 (15.6)	1 (1)	2 (2.1)	1 (1)	0	0	0
Sputum (*n* = 96)	4 (4.2)	4 (4.2)	1 (1)	4 (4.2)	5 (5.2)	1 (1)	1 (1)	0
Wound sample (*n* = 96)	8 (8.3)	5 (5.2)	2 (2.2)	3 (3.1)	4 (4.2)	3 (3.1)	0	0
Hospital wards of patients								
Intensive care unit (*n* = 42)	4 (9.8)	7 (16.7)	0	1 (2.4)	1 (2.4)	1 (2.4)	0	0
Medical (*n* = 86)	15 (17.4)	8 (14.3)	2 (2.3)	4 (4.7)	3 (3.5)	1 (1.2)	2 (2.3)	1 (1.2)
Surgical (*n* = 38)	5 (13.2)	0	1 (2.6)	1 (2.6)	3 (7.9)	1 (2.6)	0	0
Orthopedics (*n* = 31)	3 (9.7)	3 (9.7)	2 (6.5)	1 (3.2)	0	1 (3.2)	0	0
Pediatrics (*n* = 30)	1 (3.3)	2 (6.7)	0	2 (6.7)	1 (3.3)	0	0	0
NICU (*n* = 37)	1 (2.7)	8 (21.6)	0	0	0	0	0	0
OPD (*n* = 120)	6 (5)	3 (2.5)	0	1 (0.83)	2 (1.7)	1 (0.83)	1 (0.83)	0
Total (*n* = 384)	35 (9.1)	31 (8.1)	5 (1.3)	10 (2.6)	10 (2.6)	5 (1.3)	3 (0.78)	1 (0.3)

Key: NICU: neonatal intensive care unit; OPD: outpatient department.

**Table 3:** ESBL production, carbapenem resistance, and carbapenemase production profiles of *Enterobacteriaceae* species isolates among patients in Northwestern Ethiopia region, 2019.

Enterobacteriaceae species	ESBL producer *N* (%)	Carbapenem resistance *N* (%)	Carbapenemase-producer *N* (%)
*E*. *coli* (*n* = 35)	13 (37.1)	2 (5.7)	1 (2.9)
*K*. *pneumoniae* (*n* = 31)	18 (58.1)	3 (9.7)	3 (9.7)
*K. ozaenae* (*n* = 5)	1 (20)	0	0
*K. oxytoca* (*n* = 10)	3 (30)	0	0
*E*. *cloacae* (*n* = 10)	6 (60)	3 (30)	5 (50)
*Citrobacter* spp. (*n* = 5)	4 (80)	0	0
Total (*n* = 100)	**44 (44)**	**8 (8.0)**	**6 (6.0)**

**Table 4:** Antimicrobial resistance profiles of *Enterobacteriaceae* isolates from different sites of infection among patients in Northwestern Ethiopia region, 2019.

Antimicrobials	*E*. *coli* (# *T* = 35)	*K*. *pneumoniae* (# *T* = 31)	*K*. *ozaenae* (# *T* = 5)	*K. oxytoca* (# *T* = 10)	*E*. *cloacae* (# *T* = 10)	*Citrobacter* spp. (# *T* = 5)	*Proteus* spp. (#*T* = 3)	*Providencia* spp. (#*T* = 1)	Total (# *T* = 100)
	*R* %	*R* %	*R* %	*R* %	*R* %	*R* %	*R* %	*R* %	*R* %
Amoxicillin	28 (80)	30 (96.8)	5 (100)	6 (60)	10 (100)	4 (80)	3 (100)	1 (100)	87 (87)
Amoxicillin-clavulanic acid	22 (62.9)	26 (83.9)	4 (80)	6 (60)	9 (90)	4 (80)	2 (66.7)	1 (100)	74 (74)
Nitrofurantoin	8 (22.9)	12 (38.7)	2 (40)	3 (30)	6 (60)	2 (40)	1 (33.3)	0	34 (34)
Sulphamethoxazole-trimethoprim	21 (60)	20 (64.5)	4 (80)	4 (40)	7 (70)	3 (60)	2 (66.7)	1 (100)	62 (62)
Gentamicin	10 (28.6)	19 (61.3)	2 (40)	6 (60)	6 (60)	1 (20)	1 (33.3)	0	45 (45)
Chloramphenicol	8 (22.9)	14 (45.2)	1 (20)	4 (40)	6 (60)	4 (80)	1 (33.3)	0	38 (38)
Ciprofloxacin	13 (37.1)	17 (54.8)	1 (20)	3 (30)	4 (40)	2 (40)	0	0	40 (40)
Cefotaxime	14 (40)	21 (67.7)	2 (40)	6 (60)	9 (90)	4 (80)	2 (66.7)	0	58 (58)
Ceftazidime	14 (40)	22 (70.1)	4 (80)	4 (40)	8 (80)	4 (80)	1 (33.3)	0	57 (57)
Cefoxitin,	10 (28.6)	16 (51.6)	4 (80)	4 (40)	9 (90)	2 (40)	1 (33.3)	0	46 (46)
Meropenem	2 (5.7)	3 (9.7)	0	0	3 (30)	0	0	0	8 (8)
Ertapenem	1 (2.9)	3 (9.7)	0	0	2 (20)	0	0	0	6 (6)

Key: # *T*: number of isolates tested against each antimicrobial agent; *R* % percent of isolates resistance to antimicrobial agent.

## Data Availability

The finding of this study is generated from the data collected and analyzed based on the stated methods and materials. All relevant data are within the manuscript.

## Abbreviations

ATCC: American Type Culture Collection
AMR: Antimicrobial resistance
BA: Blood agar
BSI: Blood stream infection
CR: Carbapenem resistance
CP: Carbapenemase-producing
CLSI: Clinical Laboratory and Standards Institute
ESBL: Extended-spectrum beta-lactamases
FHCSH: Felege Hiwot Comprehensive Specialized Hospital
LRTI: Lower respiratory tract infections
MAC: MacConkey agar
MHA: Muller-Hilton agar
SPSS: Statistical Package for Social Sciences
UTI: Urinary tract infection.
